# Non-steroidal anti-inflammatory drug use in patients with varying severity of coronary artery disease

**DOI:** 10.1007/s00228-026-04132-5

**Published:** 2026-07-10

**Authors:** Anne Bech-Drewes, Kasper Bonnesen, Morten Böttcher, Jacob Hartmann Søby, Simon Winther, Lars Pedersen, Henrik Toft Sørensen, Timothy L. Lash, Morten Schmidt

**Affiliations:** 1 Department of Clinical Epidemiology, Center for Population Medicine, Aarhus University and Aarhus University Hospital, Olof Palmes Allé 43, Aarhus, 8200 Denmark; 2 Department of Clinical Pharmacology, Copenhagen University Hospital, Bispebjerg and Frederiksberg, Copenhagen, Denmark; 3 Department of Cardiology, Goedstrup Hospital, Herning, Denmark; 4 Department of Clinical Medicine, Aarhus University, Aarhus, Denmark; 5 Department of Epidemiology, Rollins School of Public Health, Emory University, Atlanta, USA

**Keywords:** Cardiovascular disease, Non-steroidal anti-inflammatory drugs, Drug utilisation study

## Abstract

**Purpose:**

We examined temporal trends in NSAID use among patients referred for evaluation of suspected coronary artery disease (CAD).

**Methods:**

We conducted a drug utilisation study using population-based healthcare data from Western Denmark, covering 3.3 million inhabitants. Three cohorts were categorised based on first-time diagnostic procedure for CAD assessment: coronary computed tomography angiography (CCTA; 2008–2022, *n* = 91,230), myocardial perfusion imaging (MPI; 2016–2022, *n* = 17,044), and all invasive coronary angiograms (ICA; 2008–2022, *n* = 128,327). We calculated the one-year prevalence proportion of NSAID use after the diagnostic procedures, stratified by procedure-defined CAD severity.

**Results:**

NSAID use declined over the study period, regardless of CAD severity in patients undergoing CCTA or ICA (*p* < 0.001). In 2021, the one-year prevalence of NSAID use was 17% among patients with severe CAD vs. 24% among patients without CAD diagnosed using CCTA (prevalence proportion ratio [PPR] 0.73, 95% confidence interval [CI]: 0.60–0.90). In patients undergoing MPI, 9% of those with severe CAD vs. 15% of those with no CAD used NSAIDs (PPR 0.58, 95% CI: 0.45–0.73). In patients undergoing ICA, 10% of those with severe CAD and 20% of those with no CAD used NSAIDs (PPR 0.53, 95% CI: 0.44–0.62).

**Conclusion:**

Although NSAID use among patients with CAD has decreased and is lower than among patients without CAD, it remains relatively high in those with severe CAD.

**Graphical abstract:**

**Figure:**
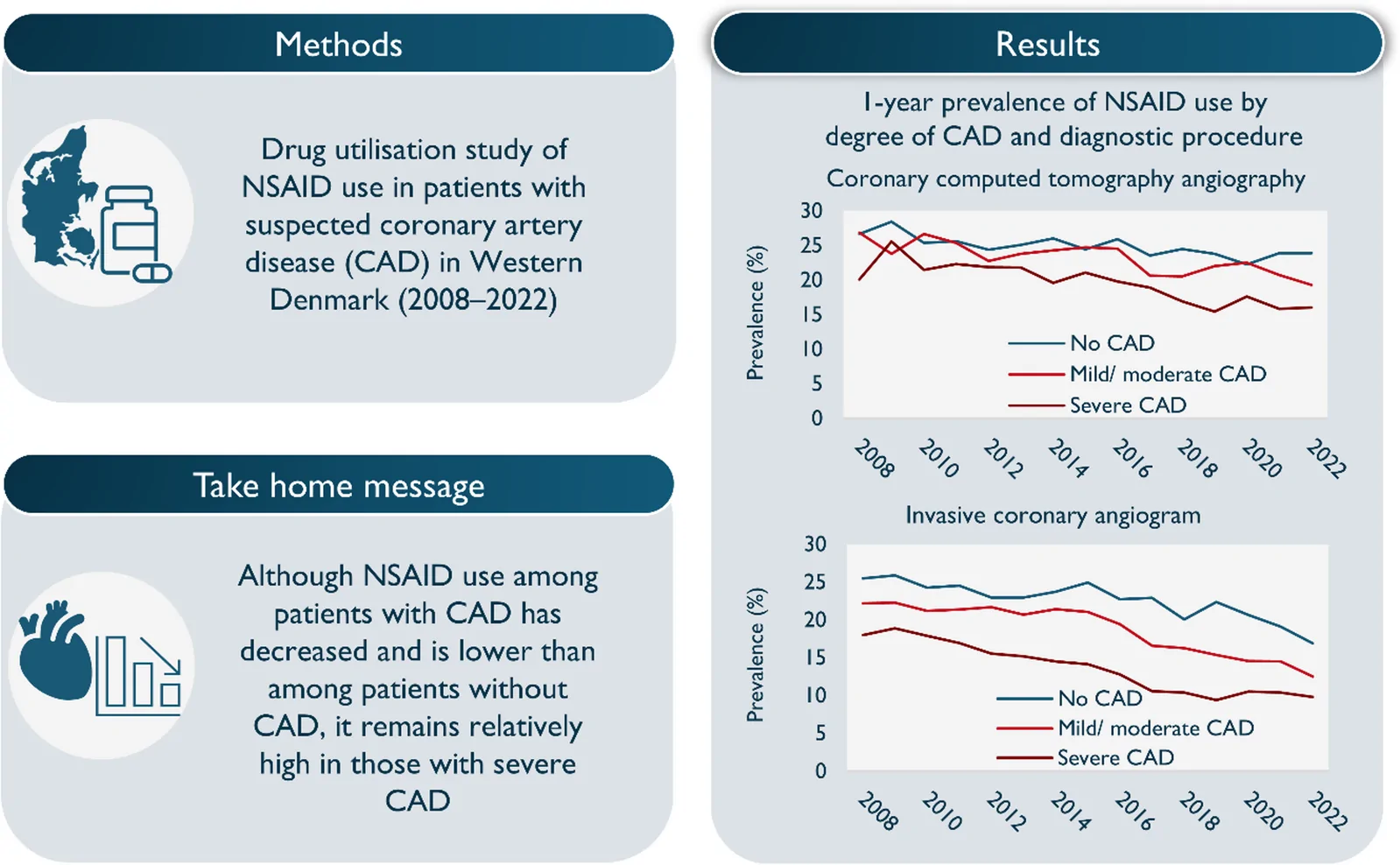

**Supplementary Information:**

The online version contains supplementary material available at https://doi.org/10.1007/s00228-026-04132-5.

## Highlights

NSAID use declined over the study period, regardless of coronary artery disease severity.

NSAID use was lower among patients with an abnormal diagnostic test for coronary artery disease than among those with a normal test.

Despite lower NSAID use among patients with abnormal test results, use remained relatively high (> 8%) in patients with severe coronary artery disease.

**Supplementary Information:**

The online version contains supplementary material available at https://doi.org/10.1007/s00228-026-04132-5.

## Introduction

Non-aspirin non-steroidal anti-inflammatory drugs (NSAIDs) are among the most widely used drugs globally due to their antipyretic, anti-inflammatory, and analgesic actions [1]. Over the past decade, several studies have highlighted the increased cardiovascular disease risk associated with NSAID use, especially in patients with preexisting cardiovascular disease [2–4]. In patients with non-obstructive coronary artery disease (CAD) confirmed with coronary computed tomography angiography (CCTA), NSAID use has been associated with a 48% increased rate of major adverse cardiac events compared to non-use [5].

Recognising these cardiovascular risks, the European Medicines Agency and the Food and Drug Administration implemented international risk minimisation strategies to discourage NSAID use in patients with cardiovascular disease [3, 6, 7]. Correspondingly, a steady decline in NSAID prescriptions among patients with newly diagnosed cardiovascular disease has been observed since 2002, particularly among patients with ischemic heart disease or heart failure [8]. Despite this decline, NSAID use continues to be substantial, for example, 14% in a recent publication in patients undergoing testing for angina pectoris [8]. For some patients, the indication may outweigh the contraindications. However, it remains unclear how NSAID use varies across patients with different severities of CAD. The aim of this study was to investigate temporal trends in the prevalence of NSAID use among patients referred for evaluation of suspected CAD.

## Methods

### Setting

The Danish National Health Service offers tax-supported universal health care, ensuring unrestricted access to hospitals and general practitioners [9]. Furthermore, patients receive partial reimbursement of the costs of prescription medications, including NSAIDs [9]. In Denmark, all citizens are issued a unique Central Personal Register number at birth or upon immigration, which is recorded in the Danish Civil Registration System [10]. This registry has tracked all changes in vital status and migration since 1968, allowing for linkage among all other Danish registries at the individual level [10].

### Study design and cohorts

We conducted a drug utilisation study using healthcare data from the Western part of Denmark (population: 3.3 million) registered in the Western Denmark Heart Registry (WDHR). The WDHR contains detailed information on all diagnostic and interventional cardiac procedures performed in the Western part of Denmark since 1999 [11, 12]. Procedures are analysed locally at the hospital where the diagnostic procedure was performed, with mandatory reporting of results to the WDHR [12]. For this study all patients examined for CAD were identified from the WDHR and categorised into three cohorts based on diagnostic procedure: non-invasive approaches comprising CCTA (January 1, 2008 to July 31, 2022), myocardial perfusion imaging (MPI) (January 1, 2016 to July 31, 2022), or invasive coronary angiograms (ICA) (January 1, 2008 to July 31, 2022) [13, 14]. Since each cohort was sampled separately, a patient may be in more than one cohort. We restricted our analysis to the first-time procedures in each cohort.

**Fig. 1:**
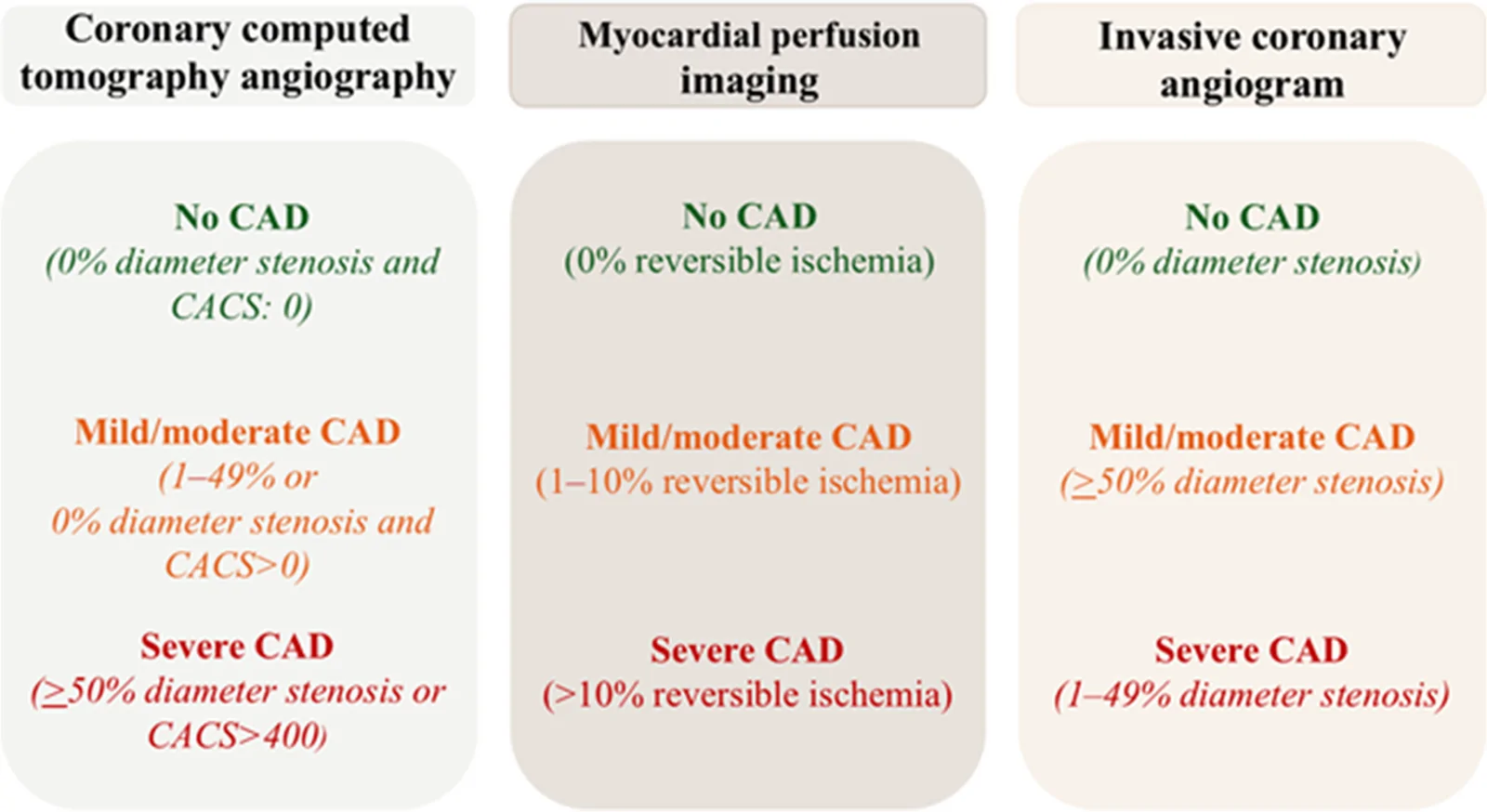
Classification of abnormal or normal tests for coronary artery disease. Abbreviation: CAD, coronary artery disease; CACS, coronary artery calcium score

CAD was classified based on CCTA, MPI, or ICA results (Fig. 1). Results for patients who underwent CCTA were categorised as having (1) no CAD (diameter stenosis: 0% and coronary artery calcium score: 0); (2) mild/moderate CAD (diameter stenosis: 1–49% or diameter stenosis: 0% and calcium score: >0); or (3) severe CAD (diameter stenosis: *≥*50% or calcium score: >400). Patients who underwent MPI were classified as having (1) no CAD (0% reversible ischemia or calcium score: 0), (2) mild/moderate CAD (1–10% reversible ischemia or calcium score: >0) or (3) severe CAD (> 10% reversible ischemia or calcium score: >400). ICA findings were grouped by diameter stenosis severity as (1) no CAD (diameter stenosis: 0%), (2) mild/ moderate CAD (diameter stenosis: 1%–49%), or (3) severe CAD (diameter stenosis: *≥*50%), with severe CAD further sub-classified by the number of diseased vessels (1–3). CAD severity was categorised using the clinical information available from each diagnostic modality.

### Non-steroidal anti-inflammatory drug use

Non-aspirin NSAIDs were identified via filled prescriptions recorded in the Danish National Prescription Registry (NPR) [15]. This registry contains nationwide information on all prescriptions filled since 1995 at Danish pharmacies, but not on medications used during hospital stays or bought over-the-counter (OTC) [16]. During the study period, low-dose ibuprofen (200 mg) was available OTC, and until December 14, 2008, diclofenac was as well [17]. The potential for identifying NSAID use from the NPR in Denmark is high [17]. As of 2012, prescription medications accounted for 92% of low-dose aspirin sales, 66% of ibuprofen sales, and 100% of all other NSAID sales [17]. Furthermore, regular users have an economic inducement to receive NSAIDs by prescription rather than OTC, as costs are partially reimbursed [9].

### Covariables

Comorbidities were identified from the Danish National Patient Registry over the 10 years preceding CCTA, MPI, or ICA, and were categorised using the Danish Index for Acute Myocardial Infarction (DANCAMI) [18, 19]. Mortality and migration data were obtained from the Danish Civil Registration System [10]. A complete list of registry codes used in the study is provided in Supplementary Table 1.

### Statistical analyses

NSAID use in 2022 across patient characteristics for each cohort is visualised in Table 1 using a heat map (Supplementary Table 2 for NSAID use in 2021). We calculated one-year prevalence proportions of NSAID use for each year and cohort, defined as the proportions of individuals with a filled NSAID prescription within one year after undergoing CCTA, MPI, or ICA. These prevalence estimates were standardised to the cohort’s age and sex distribution in 2017 for annual trend analyses [8]. To assess changes over time, a non-parametric rank-sum test for trend was applied [20]. To examine the association between CAD severity and one-year NSAID use, age- and sex-standardised prevalence proportion ratios (PPRs) with 95% confidence intervals (CIs) were calculated. Direct standardisation was performed using the age and sex distribution of patients without CAD in the same year and cohort to allow for balanced comparisons across CAD severity strata. In addition, we calculated NSAID use at predefined time points (30, 90, and 360 days) following CCTA, MPI, and ICA among new users (no NSAID use within 90 days prior) and continuous users (prescription redeemed within 90 days prior) and stratified by CAD severity to explore short- and long-term use. All analyses were stratified by sex, age, comorbidity burden, and severity of CAD. Finally, we conducted secondary analyses of NSAID subtype-specific trends and one-year cumulative NSAID use measured in defined daily doses (DDDs) and categorised as < 15, 15–50, and > 50 DDDs [8]. All analyses were conducted using STATA software V.18.0 (STATA, College Station, TX, USA).

**Table 1:**
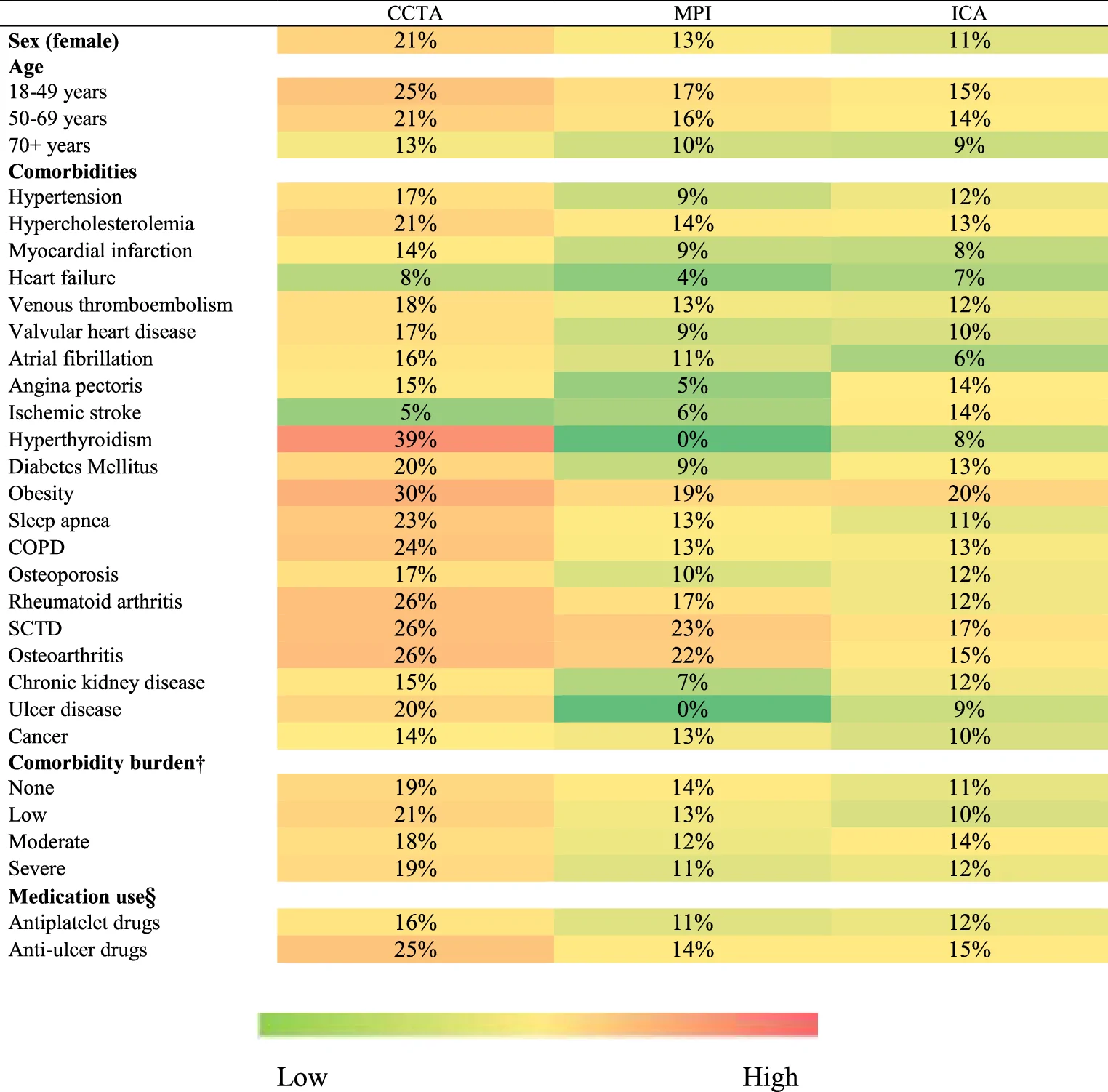
Heat map of NSAID use (%) within the first year after first-time diagnostic procedures for CAD assessment, by patient characteristics (2022)

*NSAID* non-steroidal anti-inflammatory drug, *CAD* coronary artery disease, *CCTA* coronary computed tomography angiography, *MPI* myocardial perfusion imaging, *ICA* invasive coronary angiogram, *COPD* chronic obstructive pulmonary disease, *SCTD* systemic connective tissue disease, *ACE* inhibitors, angiotensin-converting enzyme inhibitors, *ARBs* angiotensin II receptor blockers, *CCBs* calcium channel blockers, *SSRI* selective serotonin reuptake inhibitors

* Any NSAID use within 365 days after first-time diagnostic procedure

† Comorbidity burden according to the Danish Comorbidity Index for Acute Myocardial Infarction (DANCAMI)

§ Prescription filling within 90 days before first-time diagnostic procedure

## Results

### Patient characteristics

The study included 91,230 patients who had undergone CCTA, 17,044 who had undergone MPI, and 128,327 patients who had undergone ICA. The overlap between the three cohorts was 1%, defined at the individual level across the entire study period (2008–2022), such that patients who underwent CCTA, MPI, and ICA at any time during the study period were classified as overlapping (Supplementary Fig. 1). In 2022, approximately 20% of women undergoing CCTA used NSAIDs within the first year after the procedure, compared with about 10% among women in each of the two other cohorts (Table 1). Within this one-year post-procedural period, NSAID use was highest across all cohorts among patients in the youngest age group (< 50 years). A consistently higher proportion of patients with comorbidities used NSAIDs in the CCTA cohort relative to the MPI and ICA cohorts. Among patients with clinical indications for NSAID treatment, such as rheumatoid arthritis, NSAID use within one year after the procedure was 26% in the CCTA cohort, 17% in the MPI cohort, and 12% in the ICA cohort. In contrast, among patients with contraindications to NSAIDs, the one-year prevalence of use remained below 15% across all cohorts.

In 2022, irrespective of diagnostic procedure and disease severity, the majority of NSAID users within the first year after the diagnostic procedure were new users (Table 2). Specifically, 12.5% of patients with severe CAD in the CCTA cohort; 6.5% in the MPI cohort, and 8.3% in the ICA cohort were classified as new users.

**Table 2:** Prevalence of NSAID use within one year after a first-time coronary artery disease procedure (2022)

	Prevalence of NSAID use after first-time diagnostic procedure, according to coronary artery disease severity (%)								
	No CAD			Mild/moderate CAD			Severe CAD		
	0–30 days	0–90 days	0-365 days	0–30 days	0–90 days	0-365 days	0–30 days	0–90 days	0-365 days
Any procedure									
- Any NSAID use	3.6	7.8	19.7	2.8	6.6	16.2	2.5	5.2	10.9
- New users (no use 90 days before)	1.6	3.3	8.5	1.4	3.8	12.1	1.8	3.9	9.0
- Prevalent users (use 90 days before)	2.1	4.5	11.2	1.4	2.8	4.1	0.6	1.2	1.9
Coronary computed tomography angiography									
- Any NSAID use	3.8	9.5	23.4	3.1	7.1	18.1	3.1	7.1	15.4
- New users (no use 90 days before)	2.4	6.2	18.0	1.3	4.1	13.5	2.0	4.8	12.5
- Prevalent users (use 90 days before)	1.3	3.3	5.5	1.9	3.1	4.6	1.1	2.4	2.9
Myocardial perfusion imaging									
- Any NSAID use	2.1	4.8	15.0	1.5	5.9	10.7	1.8	3.2	8.5
- New users (no use 90 days before)	0.6	2.1	10.9	0.8	1.9	7.3	0.4	1.6	6.5
- Prevalent users (use 90 days before)	1.5	2.8	4.0	0.8	3.1	3.4	1.4	1.6	2.0
Invasive coronary angiogram									
- Any NSAID use	5.9	8.4	17.5	2.1	5.7	12.7	2.4	4.9	9.8
- New users (no use 90 days before)	4.3	5.7	13.0	2.0	3.9	10.0	2.1	4.2	8.3
- Prevalent users (use 90 days before)	1.6	2.7	4.6	0.2	1.8	2.7	0.3	0.7	1.5

*NSAID* non-steroidal anti-inflammatory drug, *CCTA* coronary computed tomography angiography, *MPI* myocardial scintigraphy, *ICA* invasive coronary angiogram

In the secondary analyses, ibuprofen was the most redeemed non-aspirin NSAID across all three cohorts; in 2022, the prevalence proportion of ibuprofen use was 14.9% in the CCTA cohort and 9.7% in both the MPI and ICA cohorts (Supplementary Table 3). When redeemed NSAID use was categorised by DDDs, most NSAID users redeemed 15–50 DDDs within one year after the diagnostic procedure (Supplementary Table 4).

### Temporal trends in NSAID use

#### Cohort undergoing coronary computed tomography angiography

**Fig. 2:**
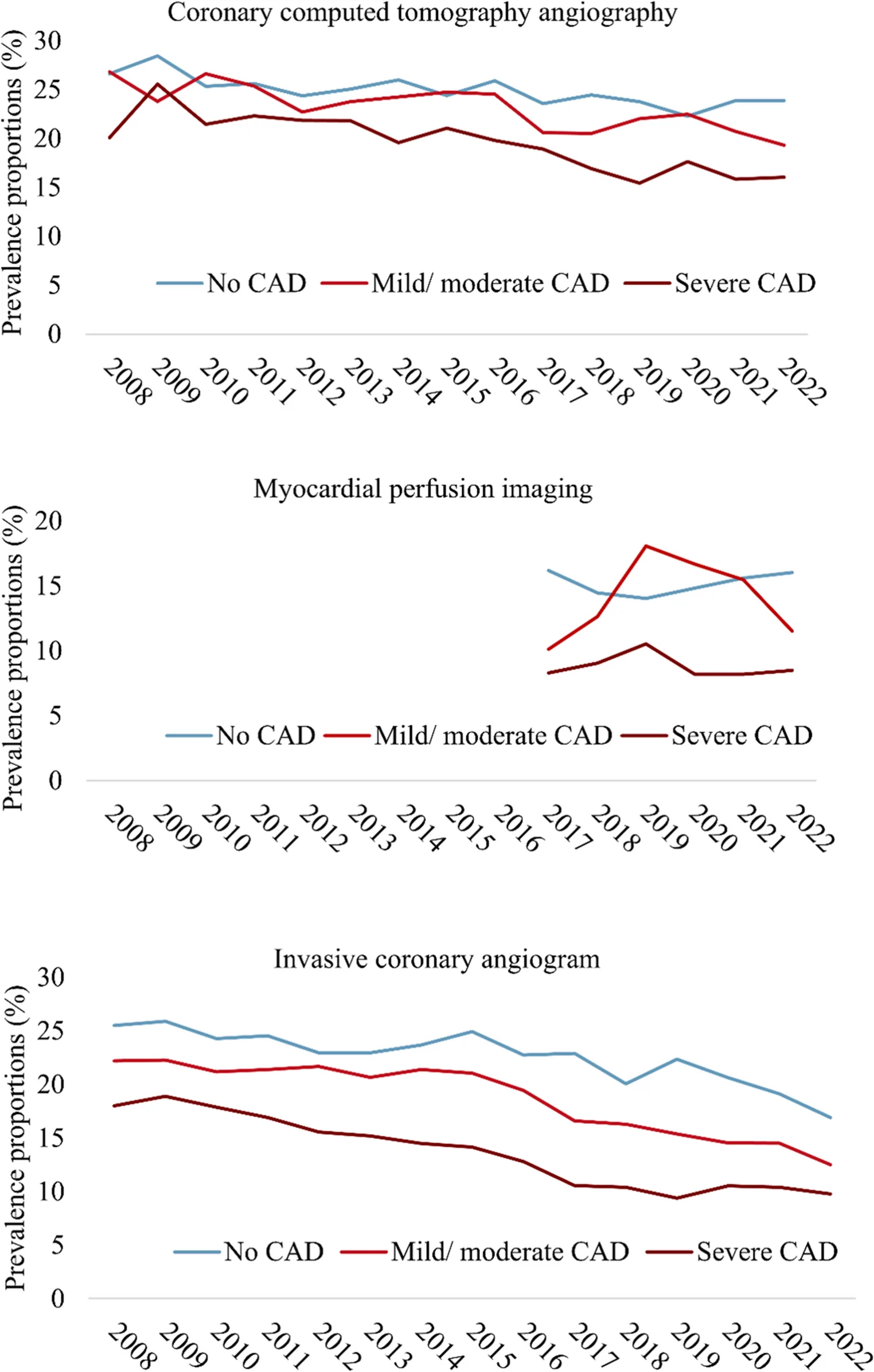
Temporal trends in the 1-year post-procedure prevalence of NSAID use by degree of coronary artery disease (CAD) and diagnostic procedure

The one-year prevalence of NSAID use within the first year after CCTA was lower among patients with severe CAD than among those with no CAD (2008, 26% vs. 28% [PPR 0.93, 95% CI: 0.49–1.78]; 2022, 19% vs. 23.5%, [PPR 0.83, 95% CI: 0.57–1.21]). For patients with severe CAD, the overall prevalence proportion for the entire study period was 20% and 24% for those with no CAD. A decline in prevalence was observed after CCTA, regardless of CAD degree (*p* < 0.001) (Fig. 2). The use was highest in patients < 49 years with an overall prevalence of 27% compared to 18.2% in patients > 70 years (Supplementary Fig. 2). Temporal trends in prevalence of NSAID use for patients who underwent CCTA remained largely unaffected by age and gender-standardisation and were unassociated with age, sex, and comorbidity burden (Supplementary Fig. 2).

#### Cohort undergoing myocardial perfusion imaging

Among patients who had undergone MPI, 26.3% had severe CAD. The prevalence of NSAID use within the first year after MPI was lower among those with severe CAD than those with no CAD (2017, 8.7% vs. 16.2% [PPR 0.53, 95% CI: 0.31–0.84]; 2022, 9.2% vs. 15% [PPR 0.62, 95% CI: 0.44–0.87]). During 2017–2021, the overall use was 8.8% among patients with severe CAD.

The NSAID use within one year after procedure was highest in 2019 among these patients, with a prevalence of 10.5% (Fig. 2). This declined to 8.5% in 2022. In 2022, the one-year prevalence was 17.4% in patients < 50 years, 16.3% in patients aged 50–69 years and 9.5% in patients > 70 years (Supplementary Fig. 3). Temporal trends in prevalence among patients who underwent MPI remained largely unaffected by stratification by age, sex, or comorbidity (Supplementary Fig. 3).

#### Cohort undergoing invasive coronary angiography

**Fig. 3:**
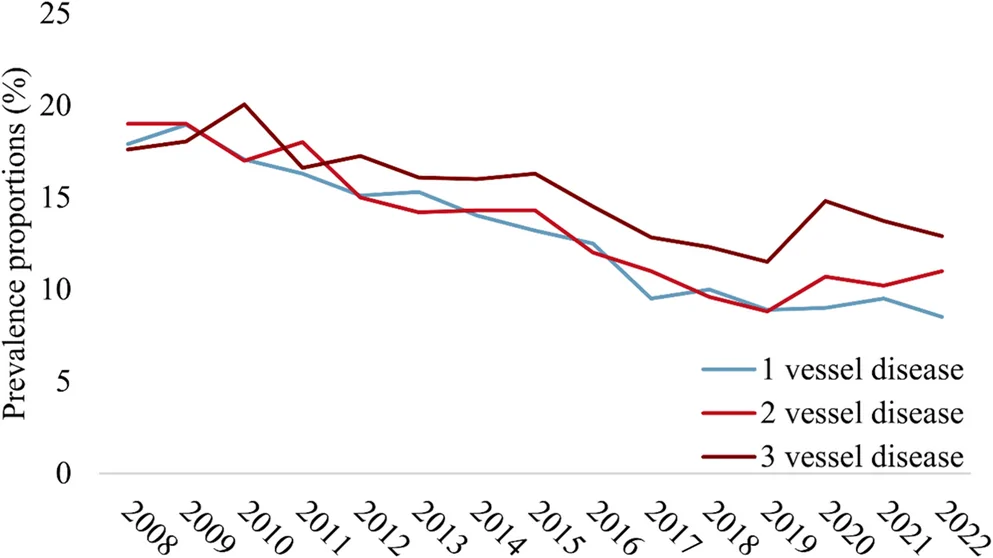
Temporal trends in 1-year prevalence of NSAID use by the number of significant diseased vessels defined by invasive coronary angiogram

The one-year prevalence of NSAID use within the first year after ICA was lower in patients with severe CAD than in those with no CAD (2008, 18.8% vs. 27% [PPR 0.70, 95% CI 0.64–0.76]; 2022, 9.9% vs. 17.5% [PPR 0.57, 95% CI: 0.44–0.73]). For patients with severe CAD, the overall prevalence proportion of NSAID use over the entire study period was lower in patients with one-vessel disease than in those with three-vessel disease (13.1% vs. 18.4%) (Fig. 3). For example, in 2022, the one-year prevalence after undergoing ICA was 8.5% in patients with one-vessel disease and 15.6% in patients with three-vessel disease (PPR 1.8, 95% CI: 1.28–2.62). Overall, NSAID use declined during the study period among patients with all degrees of diameter stenosis (*p* < 0.001) (Fig. 2). In 2022, NSAID use was most common among patients younger than 50 years, with 14.6% using NSAIDs within one year after the procedure, compared with 13.6% among those aged 50–69 years and 9.1% among those aged 70 years or older (Supplementary Fig. 5). Temporal trends in prevalence among patients undergoing ICA were consistent when stratified by age, sex or comorbidities (Supplementary Fig. 5).

## Discussion

Use of NSAIDs within one year after invasive and non-invasive diagnostic procedures for CAD was generally lower among patients with an abnormal test for CAD compared with those with a normal test. Across diagnostic modalities, patients with comorbidities had a higher prevalence of NSAID use within the first year after procedure in the CCTA cohort compared with the MPI and ICA cohorts. Furthermore, the prevalence of NSAID use following the diagnostic procedures decreased over the entire study period, regardless of CAD test results, in patients who had undergone CCTA or ICA. Nonetheless, NSAID use remained highly prevalent in patients with confirmed CAD (*≥* 8%).

### Previous literature

Few studies have examined nationwide NSAID use in patients undergoing invasive or noninvasive examination for CAD. However, the high prevalence and declining trend observed in our study align with available evidence in patients with cardiovascular disease. A Danish study examining the prevalence of NSAID initiation after a new cardiovascular diagnosis found an annual decline of close to 3% during 2002–2017 [8]. This trend was strongest for patients with heart failure and ischaemic heart disease [8]. Furthermore, a one-year NSAID prevalence of 16.6% was reported among patients with venous thromboembolism [8]. In Italy, the prevalence of NSAID use among patients with CAD ranged higher, from 21% to 48% in patients *≥* 65 years with a cerebro/cardiovascular event between 2008 and 2011 [21]. In the USA, cardiovascular patients were found to be 2.1 times more likely to use NSAIDs than those without cardiovascular disease, based on NHANES data (2009–2010) [21, 22]. Another US study also reported notably higher NSAID use compared to the general population across six cardiovascular disease cohorts. However, the trend declined from 1988 to 2016 [23]. In a 2018 Canadian study, NSAID prescriptions were frequent among high-risk patients with a history of hypertension, heart failure, or chronic kidney disease who visited primary care providers due to musculoskeletal disorders (10%) [24]. However, NSAID use declined over time, with an absolute reduction of 2.1% (2012–2016) [24].

### Limitations

A limitation of this study is the lack of information on OTC and in-hospital NSAID use, which potentially led to an underestimation of the true NSAID use. However, the tax-funded reimbursement scheme in Denmark encourages prescription use over OTC use, and in-hospital use likely constitutes only a small fraction of total use [16]. Also, NSAID use was based on actual dispensing at pharmacies and not on written prescriptions [15].

The WDHR is known for high data quality, including high accuracy [12–14, 25]. For example, the positive predictive value for the coronary artery calcium scores used to define CAD level is > 96% [13].

The pattern that patients with comorbidities had a higher prevalence of NSAID use within the first year after procedure in the CCTA cohort compared with the MPI and ICA cohorts should be interpreted with caution and may likely be associated with the differences in patient characteristics, including age, sex, and comorbidity burden.

The data originates from the Western part of Denmark only. However, the Danish population is relatively homogeneous with respect to health-related characteristics, so NSAID use is unlikely to differ substantially across the country [26]. Nevertheless, Denmark’s demographic composition may limit generalisability to more ethnically diverse populations or countries with different health care systems, prescribing practices, or NSAID availability.

### Implications

NSAID prescribing in patients with CAD may sometimes represent a clinical “Hobson’s choice”, as effective pain relief may be needed despite cardiovascular and gastrointestinal concerns [27, 28]. Nonetheless, the European Society of Cardiology advocates generally against NSAIDs use in patients with cardiovascular disease [3]. This aligns with the observed overall decline in dispensed NSAID use among patients with CAD, and the lower utilisation in patients with severe CAD compared to those without CAD. These patterns are consistent with regulatory risk-minimisation measures and safety warnings issued by the European Medicines Agency and the Food and Drug Administration discouraging NSAID use in patients with cardiovascular disease [3, 6, 7]. However, significant room for improvement remains, as the 1-year prevalence of NSAID use remains high in patients with confirmed CAD for whom NSAIDs could be contraindicated.

## Conclusions

Overall, NSAID use declined over the study period among patients examined for CAD, irrespective of disease severity. Nevertheless, NSAID use remained common in patients with significant CAD, despite clinical recommendations to restrict their use in these patients.

## Supplementary Information

Below is the link to the electronic supplementary material.

Supplementary Material 1

## Data Availability

In accordance with Danish legislation, the data cannot be made publicly available because they contain sensitive patient information.
