# Supplementary material for: Non-steroidal anti-inflammatory drug use in patients with varying severity of coronary artery disease
Source: Eur J Clin Pharmacol. 2026 Jul 10;82(8):202. doi: 10.1007/s00228-026-04132-5 (PMC13350150; doi:10.1007/s00228-026-04132-5)

**Supplementary materials**

**Supplementary Table 1**. ICD and ATC codes used in the study.

| **Variables** | **ATC codes** |
| --- | --- |
| **NSAIDs** | |
| **NSAIDs** | ATC: M01A, except M01AX05 |
| Ibuprofen | ATC: M01AE01, M01AE51 |
| Naproxen | ATC: M01AE02 |
| Ketoprofen | ATC: M01AE03, M01AE53 |
| Dexibuprofen | ATC: M01AE14 |
| Piroxicam | ATC: M01AC01 |
| Tolfenamic acid | ATC: M01AG02 |
| Diclofenac | ATC: M01AB05, M01AB55 |
| Etodolac | ATC: M01AB08 |
| Meloxicam | ATC: M01AC06 |
| **Diseases (study population, outcomes, and comorbidity) ICD-8, ICD-10, and ATC codes** | |
| Ischemic heart disease (overall) | ICD-8: 410-414; ICD-10: I121-125 |
| Hypertension | ICD-8: 400-404; ICD-10: I10-I15  or combination of ≥2 of the following ATC classes: α adrenergic blockers (C02A, C02B, C02C), non-loop diuretics (C02DA, C02L, C03A, C03B, C03D, C03E, C03X, C07C, C07D, C08G, C09BA, C09DA, C09XA52), vasodilators (C02DB, C02DD, C02DG, C04, C05), beta blockers (C07), calcium channel blockers (C07F, C08, C09BB, C09DB), and renin-angiotensin system inhibitors (C09). |
| Stable angina pectoris (chronic coronary syndrome) | ICD-8: 413; ICD-10: I20 (without I200), I251, I259 |
| Unstable angina pectoris | ICD-8: 411; ICD-10: I200 |
| Myocardial infarction | ICD-8: 410; ICD-10: I21 |
| NSTEMI | ICD-10: I211A, I210A, I214 |
| STEMI | ICD-10: I211B, I210B, I213 |
| Heart failure | ICD-8: 427.09, 427.10, 427.11, 427.19, 428.99, 782.49; ICD-10: I50.0, I50.1, I50.2, I50.3, I50.8, I50.9, I11.0, I13.0, I13.2, I42.0, I42.6, I42.7, I42.8, I42.9 |
| Cardiac arrhythmia | ICD-8: 427.20, 427.21, 427.22, 427.23, 427.93, 427.94, 427.91, 427.97; ICD-10: I440, I441, I442, I443, I455A, I455B, I455C, I455G, I470, I472, I48, I490 |
| Atrial fibrillation/flutter | ICD-8: 427.93, 427.94; ICD-10: I48 |
| Bradycardia | ICD-8: 42720, 42721, 42722, 42723; ICD-10: I440, I441, I442, I443, I455A, I455B, I455C, I455G |
| VT or VF | ICD-8: 42797, 42791; ICD-10: I470, I472, I490 |
| Cardiac arrest | ICD-8: 42727, 42797; ICD-10: I46 |
| Valvular heart disease (any) | ICD-8: 394, 395; ICD-10: I05, I34, I390, I511A, I06, I35, I391 |
| Hypercholesterolemia | ICD-8: 27200; ICD-10: E780 |
| Venous thromboembolism | ICD-8: 45100, 45108, 45109, 45099; ICD-10:1801-1803, 126 |
| Arterial claudication | ICD-8: 443.89–443.99; ICD-10: I73.9A |
| Pericarditis | ICD-8: 39109, 393, 420, 423; ICD-10: I30–I32 |
| Myocarditis | ICD-8: 422; ICD-10: I40, I41, I090, I514 |
| Endocarditis | ICD-8: 421; ICD-10: I33, I38, I398 |
| Inflammatory rheumatic disease | ICD-8: 446, 712–714, 715–716, 135.99; ICD-10: M02–03, M05–14, M30–M36, M45–46 |
| Degenerative rheumatic disease | ICD-8: 713, 725, 728; ICD-10: M15–25, M47–54 |
| Osteoarthritis | ICD-8: 713; ICD-10: M15–19 |
| Soft tissue disorders | ICD-8: 716–717; ICD-10: M65–790 |
| Osteoporosis | ICD-8: 723.09; ICD-10: M80–M82 |
| Headache | ICD-8: 791.99; ICD-10: G43–44 |
| Dementia | ICD-8: 290.09-290.19, 293.09; ICD-10: F002-F03, F05.1, G30 |
| Cancer | ICD-8: 140–207; ICD-10: C00–C96 |
| Obesity | ICD-8: 277; ICD-10: E65–E68 |
| Hyperthyroidism | ICD-8: 242; ICD-10: E05 |
| Sleep apnea | ICD-10: G47.3 |
| Ulcer disease | ICD-8: 530.91, 530.98, 531–534; ICD-10: K22.1, K25–K28 |
| Schizophrenia | ICD-10: F20–F22, F25, F28, F29; ATC: N05A |
| Diabetes mellitus | ICD-8: 249–250; ICD-10: E10–E14, O24 (except O24.4), H36.0; ATC: A10B, A10A |
| COPD | ICD-8: 491–492; ICD-10: J41–44; ATC: R03 |
| Chronic kidney disease | ICD-8: 249.02, 250.02, 753.10–753.19, 582, 583, 584, 590.09, 593.20, 792; ICD-10: E10.2, E11.2, E14.2, N03, N05, N11.0, N14, N16, N18-N19, N26.9, Q61.1–Q61.4 |
| Stroke overall | ICD-10: I60–64 |
| Alcoholism-related diseases | ICD-8: 291, 303, 456, 571.09, 571.10, 577.10; ICD-10: F10.1-9, G31.2, G62.1, G72.1, I 42.6, K29.2, K86.0, Z72.1;  ATC: N07BB |
| Chronic liver disease | ICD-8: 70.00, 70.02, 70.04, 70.06, 70.08, 573.00, 456.00-456.09, 571; 573.01, 573.04; ICD-10: B18; K70.0-K70.3; K70.9; K71; K73; K74; K76.0, B15.0; B16.0; B16.2; B19.0 |
| DANCAMI* |  |
| Congestive heart failure | ICD-10: 150, I11.0, I13.0, I12.2 |
| Intermittent arterial claudication | ICD-10: 173.9 |
| Aorta disease | ICD-10: I7.1 |
| Heart valve disease | ICD-10: I05–I09, I34–I39 |
| Stroke | ICD-10: I60, I61, I64 |
| Hypertension | See codes for hypertension above |
| High risk cancer | ICD-10: C13, C15, C16, C22–C26, C33, C45, C77–C79, C92.3–C92.9, C95 |
| Lower-risk cancer | ICD-10: C00–C12. C14, C17–C21, C30–C32, C37–C44, C46–C76, C80–C91, C92.1, C93, C96, C97 |
| Coagulopathy and other blood disorders | ICD-10: D55–D61, D63, D64, D66–D72, D74–D77. |
| Diabetes mellitus without end-organ damage | ICD-10: E10.0, E10.1, E10.9, E11.1, E11.9, E12.0, E12.1, E12.9, E13.0, E13.1, E13.9, E14.0, E14.1, E14.9; ATC A10A |
| Diabetes mellitus with end-organ damage  Dementia  Alcohol and drug abuse  Schizophrenia  Affective disorder  Epilepsy  Atrophy, degenerative disease or demyelination of CNS  Chronic kidney disease  Chronic pulmonary disease  Ulcer  Mild liver disease  Moderate to severe liver disease  Chronic pancreatitis | ICD-10: E10.2–E10.8, E11.2–E11.8, E12.2–E12.8, E13.2–E13.8, E14.2–E14.8, H36.0  ICD-10: F00–F03, F05.1, G30  ICD-10: F10–F19, Z50.2, Z50.3, Z71.4, Z71.5  ICD-10: F20–F22, F25, F28, F29; ATC: N05A  ICD-10: F30–F34, F38, F39; ATC: N06A  ICD-10: G40, G41  ICD-10: G10–G13, G20–G23, G25.5, G31.2, G31.8, G31.9, G35–G37, G90, G93.4  ICD-10: E10.2, E11.2, E14.2, I12, DI13, N03, N05, N11.0, N14, N16, N18, N19, N26.9, Q61.1–Q61.4, Z99.2  ICD-10: J40–J47, J60–J67, J68.4, J70.1, J70.3, J84.1, J92.0, J96.1, J98.2, J98.3; ATC: R03  ICD-10: K22.1, K25–K28  ICD-10: B81, K70.1–K70.3, K70.9, K71, K73, K74, K76.0  ICD-10: B15.0, B16.0, B16.2, B19.0, K70.4, K72, K76.6, I85  ICD-10: K86.0, K86.1 |
| Comedications | |
| Angiotension converting enzyme (ACE) inhibitors | ATC: C09AA, C02EA |
| Angiotension 2 receptor Blockers (ARBs) | ATC: C09CA, C02EX01 |
| Beta-blockers | ATC: C07 |
| Calcium channel blockers | ATC: C08 |
| Diuretics | ATC: C03 and C02DF01 |
| Nitrates | ATC: C01DA |
| Statins | ATC: C10AA, C10B, B04AB |
| Antiplatelet drugs | ATC: B01AC, N02BA01 |
| Anticoagulant drugs | ATC: B01AA, B01AB, B01AE, B01AF |
| SSRIs | ATC: N06AB |
| Antipsychotic drugs | ATC: N05A |
| Anti-ulcer drugs | ATC: A02B |
| Systemic glucocorticoids | ATC: H02AB |
| Gout agents | ATC: M04A |
| Methotrexate | ATC: L01BA01, L04AX03 |
| Paracetamol | ATC: N02BE01 |
| Opiods | ATC: N02A |

* Comorbidity burden according to the Danish Comorbidity Index for Acute Myocardial Infarction (DANCAMI).

| **Supplementary Table 2**. Heat map displaying NSAID use (%) across characteristics for each cohort (2021) | | | |
| --- | --- | --- | --- |
|  | CCTA | MPI | ICA |
| Sex (female) | 21% | 13% | 11% |
| Age |  |  |  |
| 18-49 years | 25% | 20% | 18% |
| 50-69 years | 22% | 17% | 11% |
| 70+ years | 13% | 10% | 8,5% |
| Comorbidities |  |  |  |
| Hypertension | 18% | 10% | 12% |
| Hypercholesterolemia | 19% | 13% | 13% |
| Myocardial infarction | 9% | 11% | 8% |
| Heart failure | 6% | 6% | 7% |
| Venous thromboembolism | 17% | 20% | 6% |
| Valvular heart disease | 19% | 14% | 10% |
| Atrial fibrillation | 12% | 8% | 8% |
| Angina pectoris | 23% | 10% | 13% |
| Ischemic stroke | 10% | 11% | 8% |
| Hyperthyroidism | 26% | 30% | 13% |
| Diabetes Mellitus | 22% | 11% | 13% |
| Obesity | 29% | 18% | 19% |
| Sleep apnea | 27% | 19% | 15% |
| COPD | 22% | 16% | 11% |
| Osteoporosis | 31% | 16% | 9% |
| Rheumatoid arthritis | 12% | 15% | 11% |
| SCTD | 22% | 16% | 19% |
| Osteoarthritis | 29% | 8% | 18% |
| Chronic kidney disease | 10% | 5% | 3% |
| Ulcer disease | 8% | 5% | 5% |
| Cancer | 19% | 15% | 9% |
| Comorbidity burden† |  |  |  |
| None | 20% | 13% | 14% |
| Low | 21% | 14% | 13% |
| Moderate | 21% | 13% | 9% |
| Severe | 20% | 10% | 10% |

Abbreviations: CCTA, coronary computed tomography angiography; MPI, myocardial perfusion imaging; ICA, invasive coronary angiogram; COPD, chronic obstructive pulmonary disease; SCTD, systemic connective tissue disease; ACE inhibitors, angiotensin-converting enzyme inhibitors; ARBs, angiotensin II receptor blockers; CCBs, calcium channel blockers; SSRI, selective serotonin reuptake inhibitors.

* Any non-steroidal anti-inflammatory drug use within 365 days after first-time diagnostic procedure.

† Comorbidity burden according to the Danish Comorbidity Index for Acute Myocardial Infarction (DANCAMI).

§ Prescription filling within 90 days before first time diagnostic procedure.

**Supplementary Figure 1:** Venn diagram illustrating the overlap of patients between the three cohorts included in the study from 2008 to 2022.

**
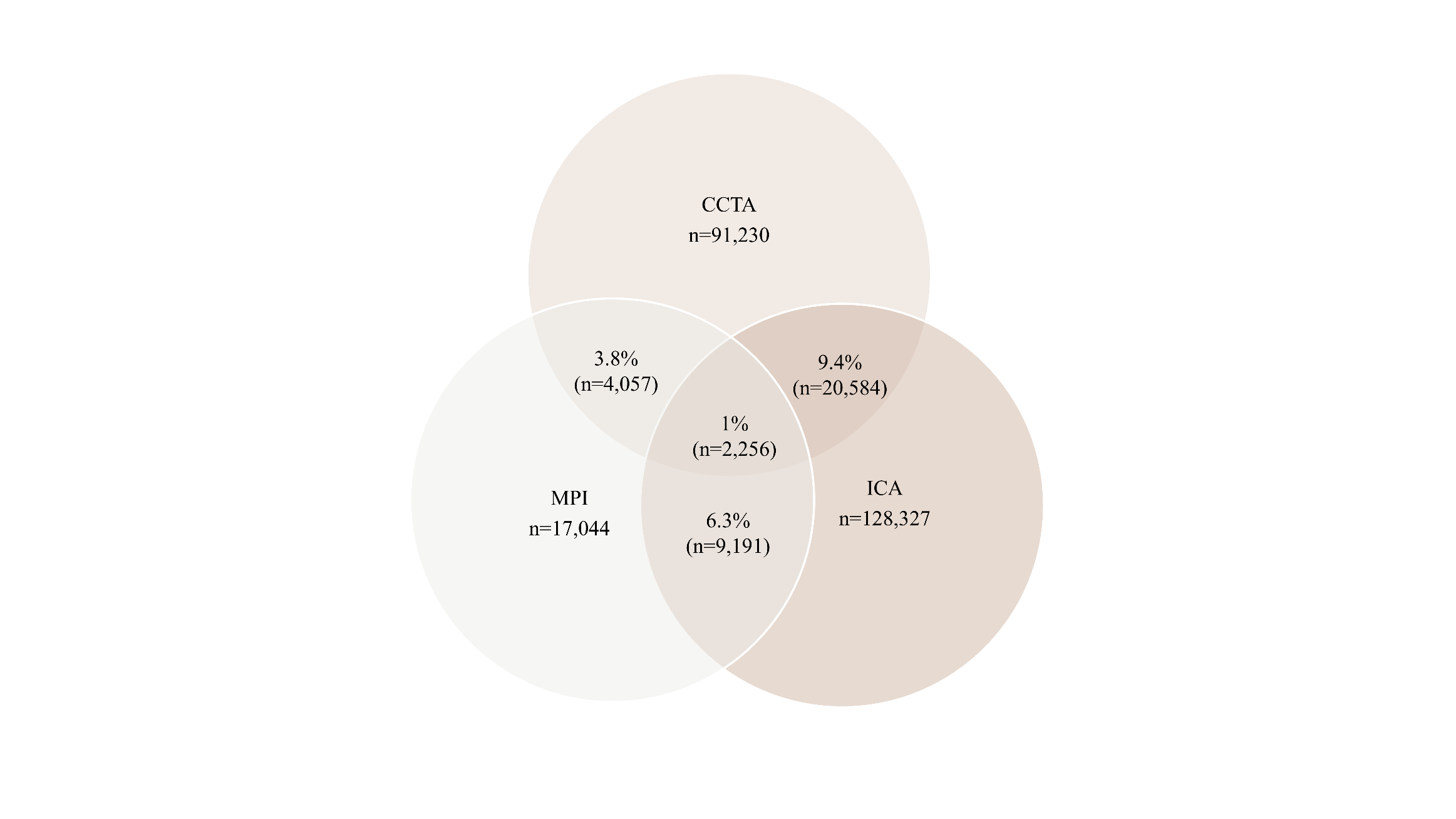
**

Abbreviations: CCTA, coronary computed tomography angiography; MPI, myocardial scintigraphy ICA, invasive coronary angiogram

**Supplementary Table 3.** Temporal trends in 1-year prevalence of non-aspirin non-steroidal anti-inflammatory drug (NSAID) use in each cohort (2008–2022).

| Prevalence of NSAID use after first-time diagnostic procedure (%) | | | | | | | | | | | | | | | |
| --- | --- | --- | --- | --- | --- | --- | --- | --- | --- | --- | --- | --- | --- | --- | --- |
|  | 2008 | 2009 | 2010 | 2011 | 2012 | 2013 | 2014 | 2015 | 2016 | 2017 | 2018 | 2019 | 2020 | 2021 | 2022 |
| Coronary computed tomography angiography | | | | | | | | | | | | | | | |
| Ibuprofen | 14.8 | 16.6 | 16.8 | 17.3 | 17.4 | 18.4 | 18.3 | 18.1 | 17.7 | 15.7 | 15.8 | 15.8 | 15.6 | 14.9 | 14.9 |
| Naproxen | 8.0 | 6.1 | 5.5 | 4.5 | 3.1 | 2.8 | 2.2 | 2.2 | 2.0 | 1.8 | 1.7 | 1.5 | 1.7 | 1.5 | 1.6 |
| Diclofenac | 1.0 | 1.1 | 0.7 | 1.4 | 1.4 | 1.3 | 1.3 | 1.1 | 1.4 | 1.8 | 1.2 | 1.1 | 1.0 | 1.1 | 0.9 |
| Myocardial perfusion imaging | | | | | | | | | | | | | | | |
| Ibuprofen | - | - | - | - | - | - | - | - | - | 10.8 | 10.3 | 10.4 | 10.5 | 10.3 | 9.7 |
| Naproxen | - | - | - | - | - | - | - | - | - | 1.2 | 1.5 | 1.2 | 1.5 | 1.4 | 1.2 |
| Diclofenac | - | - | - | - | - | - | - | - | - | 0.7 | 0.9 | 1.0 | 0.6 | 0.6 | 1.0 |
| Invasive coronary angiogram | | | | | | | | | | | | | | | |
| Ibuprofen | 13.1 | 13.9 | 14.4 | 14.5 | 14.1 | 14.1 | 14.5 | 14.5 | 13.1 | 11.7 | 10.8 | 10.9 | 10.1 | 10.5 | 9.7 |
| Naproxen | 5.3 | 4.9 | 4.1 | 3.4 | 2.8 | 2.1 | 1.7 | 1.8 | 1.6 | 1.3 | 1.2 | 0.9 | 1.1 | 1.0 | 1.2 |
| Diclofenac | 0.9 | 0.9 | 0.9 | 0.9 | 1.3 | 1.0 | 1.2 | 1.1 | 1.1 | 1.0 | 0.9 | 0.8 | 0.6 | 0.6 | 0.4 |

**Supplementary Table 4.** Annual trends in defined daily dose categories* of non-steroidal anti-inflammatory drug (NSAID) use within one year after diagnostic procedure, by cohort (2008–2022).

| Prevalence of NSAID use in the defined daily dose categories (%) | | | | | | | | | | | | | | | |
| --- | --- | --- | --- | --- | --- | --- | --- | --- | --- | --- | --- | --- | --- | --- | --- |
|  | 2008 | 2009 | 2010 | 2011 | 2012 | 2013 | 2014 | 2015 | 2016 | 2017 | 2018 | 2019 | 2020 | 2021 | 2022 |
| Coronary computed tomography angiography | | | | | | | | | | | | | | | |
| Low use | 2.5 | 3.7 | 3.3 | 3.5 | 3.3 | 3.2 | 3.2 | 3.2 | 3.2 | 3.1 | 3.5 | 3.8 | 3.3 | 3.5 | 3.3 |
| Moderate use | 11.3 | 9.5 | 10.3 | 9.4 | 8.9 | 9.4 | 9.1 | 9.3 | 9.5 | 8.2 | 8.7 | 7.9 | 8.0 | 8.0 | 7.9 |
| High use | 12.1 | 12.2 | 11.1 | 11.2 | 8.9 | 10.3 | 9.9 | 9.3 | 8.5 | 7.4 | 6.6 | 6.7 | 6.8 | 5.9 | 6.0 |
| Myocardial perfusion imaging | | | | | | | | | | | | | | | |
| Low use | - | - | - | - | - | - | - | - | - | 4.3 | 3.3 | 2.6 | 2.9 | 2.8 | 2.6 |
| Moderate use | - | - | - | - | - | - | - | - | - | 5.9 | 5.4 | 5.4 | 5.7 | 5.3 | 5.3 |
| High use | - | - | - | - | - | - | - | - | - | 2.8 | 5.0 | 4.6 | 4.3 | 4.5 | 4.3 |
| Invasive coronary angiogram | | | | | | | | | | | | | | | |
| Low use | 3.2 | 3.1 | 3.2 | 2.8 | 2.8 | 2.8 | 2.9 | 2.8 | 3.0 | 2.8 | 3.4 | 3.1 | 3.1 | 3.0 | 2.6 |
| Moderate use | 8.4 | 8.2 | 7.6 | 7.9 | 7.6 | 6.9 | 7.3 | 7.8 | 7.3 | 5.9 | 5.7 | 5.7 | 5.8 | 5.7 | 5.8 |
| High use | 9.4 | 10.2 | 9.6 | 9.1 | 8.5 | 8.5 | 7.6 | 7.3 | 5.7 | 5.3 | 4.4 | 3.9 | 3.8 | 3.5 | 3.0 |

*Cumulative NSAID use within one year after the diagnostic procedure was categorised as low (<15 DDDs), moderate (15–50 DDDs) and high (>50 DDDs).

**Supplementary Figure 2.** Temporal trends in 1-year prevalence of NSAID use after first-time coronary computed tomography angiography (A), myocardial perfusion imaging (B) and invasive coronary angiogram (C), according to age, sex, and comorbidity burden.

A. Coronary computed tomography angiography


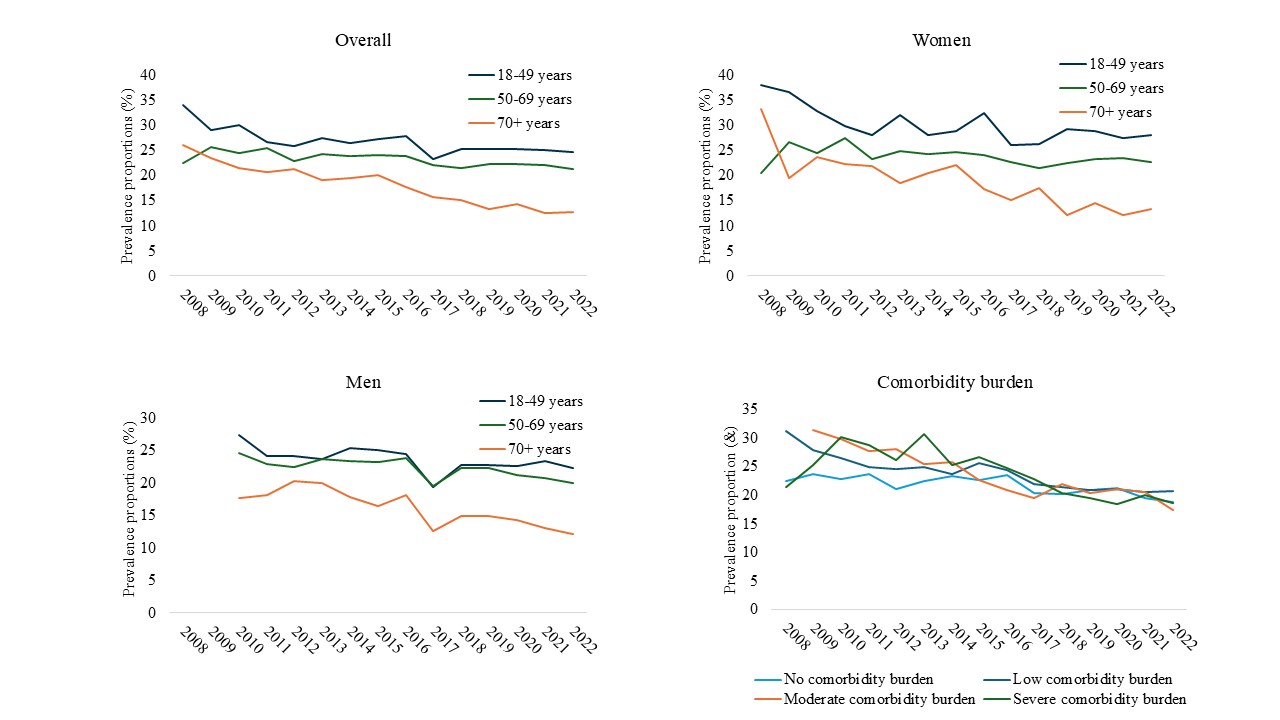


B. Myocardial perfusion imaging
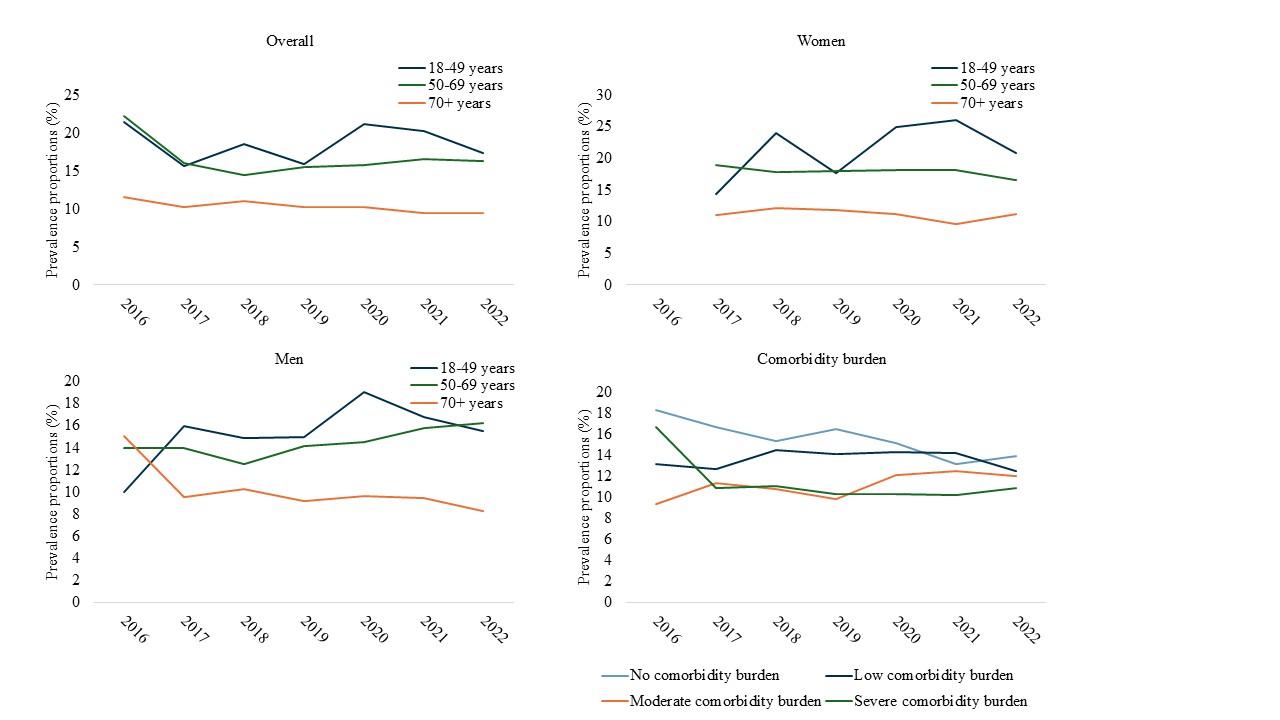


C. Invasive coronary angiogram


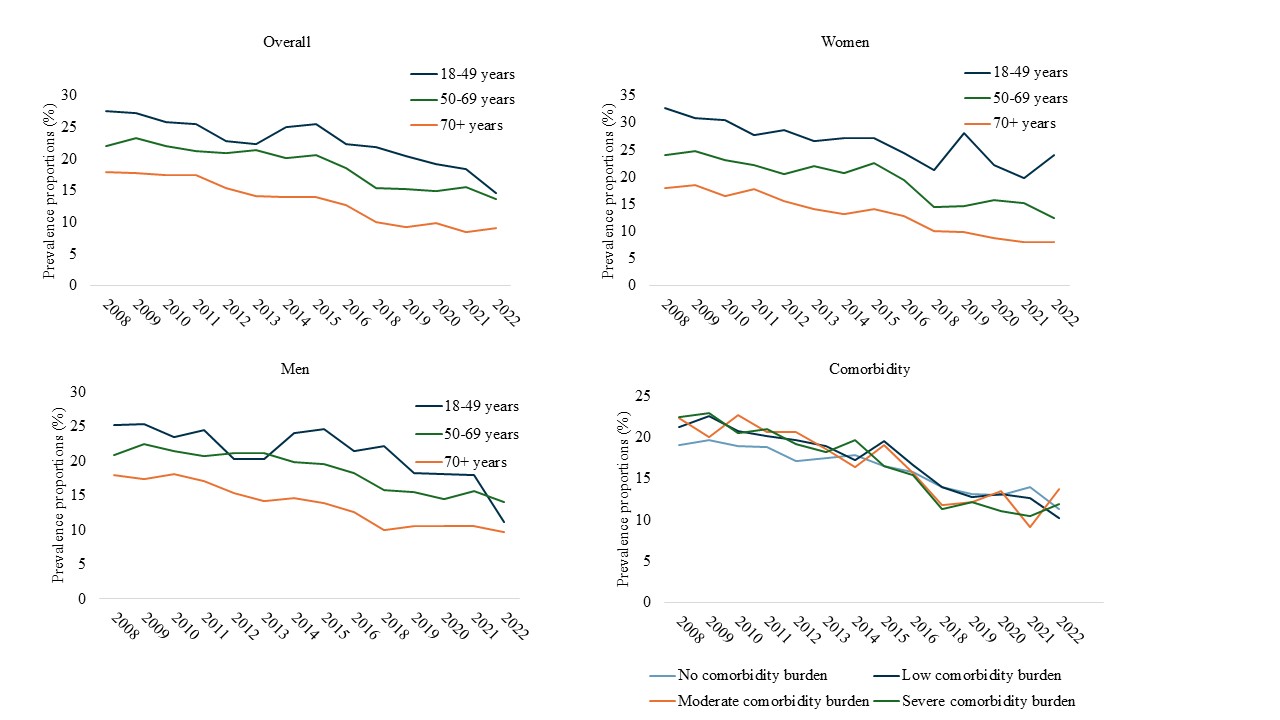

Supplement: Supplementary file 1 — Supplementary Material 1 [file 228_2026_4132_MOESM1_ESM.docx]
